# The Meta‐Analytic Evidence Is In—Time to Get on and Improve Our Treatments

**DOI:** 10.1002/eat.24520

**Published:** 2025-08-08

**Authors:** Tracey D. Wade

**Affiliations:** ^1^ Flinders Institute for Mental Health and Wellbeing, Blackbird Initiative Flinders University Adelaide Australia

**Keywords:** active comparators, adaptive trials, cognitive behavior therapy, meta‐analysis

## Abstract

The good news is that Bruns and colleagues' robust meta‐analysis of randomized controlled trials (RCTs) of individual Cognitive‐Behavioral Therapy (CBT) for eating disorders has provided us with evidence congruent with other recent meta‐analyses in this area. The emerging message, however, holds up an unflattering mirror reflecting the following regarding the use of CBT with eating disorders; we do not know whether to use CBT with adults who have anorexia nervosa; CBT is better than doing nothing with the other eating disorder diagnostic groups; any form of therapist input will suffice as the length and intensity of the CBT make no difference to outcomes; all evidence‐based therapeutic approaches seem to perform just as well as CBT. The field needs to rise to the challenge to offer something more informative for clinicians and consumers alike, and three research strategies to achieve this are described. Evidence‐based approaches to improving overall outcomes of all our therapies for eating disorders are also described. The use of these approaches in our existing therapies can be evaluated to examine whether these achieve improved remission rates. The challenge for our research community is not in producing further meta‐analyses but in improving CBT for people with eating disorders.

## 1 The Good News

Bruns et al. (2025) meta‐analysis of 41 randomised controlled trials (RCTs) of cognitive behaviour therapy (CBT) for eating disorders joins a recent influx of meta‐analyses examining the efficacy of CBT with eating disorders. Five have appeared over the last year ‐ four in 2024 (two of which focused on internet CBT) and one network meta‐analysis in 2025.

The good news is that a common story is emerging across these meta‐analyses (Bruns et al. 2025; Cuijpers et al. 2024, 2025; Öst et al. 2024), all of which are robust and pay special attention to methodological issues that could otherwise reduce confidence in the results obtained. First, we do not have enough good quality studies to reliably inform us about the efficacy of CBT for individual categories of eating disorders, but there is a particular paucity of such studies for anorexia nervosa. Second, the between‐group effect sizes (ES) with wait list conditions (not used in anorexia nervosa) range from 0.76 to 1.06. Third, overall, the evidence suggests no significant differences between CBT and treatment as usual (TAU) or active intervention controls. Fourth, effectiveness studies have very similar within‐group ES for reductions in disordered eating as efficacy studies, around 1.20 by the end of treatment (a mean of 27 sessions), like the reductions observed for 10‐session CBT, an ES of 1.49 (Keegan et al. 2022). Fifth, there are no significant differences across the effects of individual, group, and guided self‐help formats of CBT. Sixth, examination of an important dichotomous indicator of clinical significance, abstinence, indicates that 36% of participants in CBT conditions versus 10% in control conditions were abstinent after treatment.

## 2 The Not‐So‐Good News

The not‐so‐good news is that these results don't really help clinicians make decisions about how to conduct their practice in the clinic. The guidance we can offer is as follows: we don't know whether to use CBT with adults who have anorexia nervosa; CBT is certainly better than doing nothing with the other eating disorder diagnostic groups, producing large effect size improvements in eating disorder pathology, moderate effect size improvements for objective binge episodes, and small effect size improvements in self‐esteem, depression, anxiety and quality of life (Cuijpers et al. 2024); any form of therapist input will suffice as the length and intensity of the CBT makes no difference to outcomes, if we accept with some optimism that “more intensive and guided treatment formats tended to show a more consistent effect than unguided treatment formats” (Bruns et al. 2025). Ultimately, we are unable to particularly recommend that they offer CBT, as all evidence‐based therapeutic approaches seem to perform the same.

## 3 A Three‐Pronged Plan to Improve CBT for Eating Disorders

It seems that we may be at the point where we do not need further meta‐analyses. Rather, we need more robust clinical trials that can progress our understanding of how to provide more effective individual CBT to people with eating disorders such that: (1) it may start to outperform other various therapeutic modalities, and (2) we know which form of CBT is best for whom. To achieve this, there are three pathways forward to consider in terms of future clinical trials.

### 3.1 Use Active Interventions as the Comparator Condition

First, waiting lists as comparators are really fit for use only in small pilot or feasibility trials. RCTs need to routinely use TAU or active interventions. Cuijpers et al. (2024) argue that waitlists do not allow us to examine the effects of therapies over the longer term, and that they may overestimate the effects of treatments compared to other control groups. Additionally, we can argue that an active intervention will permit examination of which therapy works best for whom, allowing us to start to develop some sophistication with treatment personalization. Our work (Wade et al. 2021) showed that less motivated people starting CBT did as well as the more motivated people, and significantly better than less motivated people, when they were randomized to receive some initial motivational interviewing content. Hardly rocket science (I hear you say) but at least such designs keep us focused on the idea that CBT needs to be adapted to suit our patient, rather than a perpetual “rolling out a one‐protocol‐fits‐all approach” which occurs when we compare stock standard CBT to waitlist conditions in RCTs. While it is challenging to develop an active comparison condition that controls for common factors but does not contain any of the active ingredients of CBT, dismantling studies such as that described above may be more feasible. Independent of active comparator interventions, work on “which therapy works best for whom” will also benefit from tackling a major barrier, namely introducing standardized assessments across clinical studies that would allow combination of data for sufficiently powered analyses. Recommendations for harmonization of data collection exist (Austin et al. 2023), and researchers and clinicians in the United Kingdom (Jewell et al. 2025) and Australia (Trompeter et al. 2021) are actively pursuing collaborative analysis of data across treatment centers.

### 3.2 Increase Use of Innovative Trial Designs

Second, and continuing the theme of treatment personalization, we need to increase our use of innovative trial designs, including individually randomized, cluster randomized, non‐inferiority, and randomizations at multiple time points that depend on the participants' intermediate outcomes or treatment responses at that time point (Ryan et al. 2024). An average of 35% of people receiving CBT achieve at least a 50% reduction in their symptoms, but this figure is associated with a high level of heterogeneity (Cuijpers et al. 2024). Trial designs that allow us to respond to the high level of individual variation observed in treatment, by building in personalization of the therapy as it progresses, work with this heterogeneity rather than seeing it as an inconvenience. Where we see evidence of gradual rather than rapid response, we can introduce a priori planned strategies to give our patients a better chance to have a good outcome. By intervening early with gradual responders and helping them benefit as much from therapy as the rapid responders, we can overall improve our CBT outcomes.

Use of such adaptive trials evaluating CBT for people with anorexia nervosa may be a particularly good investment of time that will help us understand how we can best improve therapy for this group. This is a population that is particularly difficult to entice into RCTs. For adolescents, both families and services seem reluctant for children to be randomized away from family‐based treatment (FBT). Of 18 RCTs examining FBT against a comparator, only four use individual psychotherapies (one of which was CBT) while the remainder used different forms of FBT (Austin et al. 2025). Adults with anorexia nervosa are also reluctant to be randomized, and service providers are likewise unwilling for randomization to dictate the treatment received. This was illustrated in the United Kingdom DAISIES trial (Phillips et al. 2023), comparing inpatient and stepped‐care day patient treatment for adults with severe anorexia nervosa.

### 3.3 Consider Routinely Reducing the Sessions in Our CBT

Third, we need to critically evaluate whether we need 20‐ and 40‐session forms of CBT for eating disorders for non‐underweight and underweight presentations, respectively. Consistent with the meta‐analytic evidence showing shorter forms of guided self‐help CBT are as effective as therapist‐led forms of CBT, it may be that shorter versions of CBT provide similar outcomes as longer versions. For example, while no direct comparisons of 10‐ and 20‐session therapist‐led CBT have been conducted, within‐group effect sizes suggest commensurate improvements (Öst et al. 2024). Shorter versions would allow for more rapid generation of knowledge of which forms of CBT are best for whom. The development of shorter versions will be assisted by a better characterization of the dynamic patterns of symptom change across different treatment protocols to better clarify which early components of treatment produce the most change, and the changes on standardized measures which best predict outcome in the early stages of therapy (Wade et al. 2025).

## 4 Improving Our Therapies for Eating Disorders

Moving to the bigger picture, it is good news for people with eating disorders that there are a range of therapies that they can access that will result in significant improvements to disordered eating. Less heartening is that only 36% of people doing some form of evidence‐based outpatient therapy for an eating disorder that is not anorexia nervosa can expect to be abstinent from eating disorder behaviors at the end of treatment (Cuijpers et al. 2024). It is my strong opinion that we need to, and can, improve that figure, regardless of which therapy we are offering.

Meta‐analytic data suggests various ways in which the outcomes of any therapy across different psychological disorders can be significantly improved, each associated with a small ES (Wade and Waller 2025). First, stratifying people to the most appropriate level of therapy for their presenting symptoms rather than following a stepped care approach. Second, personalization of therapy, which involves tailoring treatment protocols to individual patients, based on their unique symptom profiles. Third, using the individual's choice of therapeutic elements to include in their therapy produces significant improvements over and above the therapist making such decisions. Fourth, push for early behavioral change in therapy. Fifth, measure symptoms at each session of therapy and share this feedback with your patient. Sixth, consider the use of theoretically appropriate acute (short) augmentations of therapy.

Across many of these strategies, there is limited information about the impact on eating disorders, as most of the data has been collected with depression and/or anxiety. It is recommended that researchers incorporate and evaluate these methods in future clinical eating disorder trials.

## 5 Concluding Remarks

Now that we are richly endowed with meta‐analytic information, it seems timely to press the pause button on further meta‐analyses of CBT for eating disorders. Our effort is better spent on devising and testing ways to improve CBT that can produce better outcomes for the variety of people who are willing to partner with a therapist to engage with a treatment for an eating disorder.

## Author Contributions

**Tracey D. Wade:** conceptualization, writing – original draft, writing – review and editing.

## Disclosure

Tracey Wade receives royalties from a therapist manual advocating for briefer cognitive behavior therapy—Waller G, Turner H, Tatham M, Mountford VA, Wade TD. (2019). Brief Cognitive Behavioral Therapy for Non‐Underweight Patients: CBT‐T for Eating Disorders. Abingdon: Routledge, Taylor & Francis Group.

## Conflicts of Interest

The author declares no conflicts of interest.

## Data Availability

Data sharing not applicable to this article as no datasets were generated or analysed during the current study.
